# Modelling song popularity as a contagious process

**DOI:** 10.1098/rspa.2021.0457

**Published:** 2021-09

**Authors:** Dora P. Rosati, Matthew H. Woolhouse, Benjamin M. Bolker, David J. D. Earn

**Affiliations:** ^1^ Department of Mathematics and Statistics, McMaster University, Hamilton, Ontario, Canada L8S 4K1; ^2^ School of the Arts, McMaster University, Hamilton, Ontario, Canada L8S 4K1; ^3^ McMaster Institute for Music and the Mind, McMaster University, Hamilton, Ontario, Canada L8S 4K1; ^4^ Department of Biology, McMaster University, Hamilton, Ontario, Canada L8S 4K1

**Keywords:** song popularity, song downloads, infectious disease, mathematical epidemiology, susceptible–infectious–recovered model, contagious processes

## Abstract

Popular songs are often said to be ‘contagious’, ‘infectious’ or ‘viral’. We find that download count time series for many popular songs resemble infectious disease epidemic curves. This paper suggests infectious disease transmission models could help clarify mechanisms that contribute to the ‘spread’ of song preferences and how these mechanisms underlie song popularity. We analysed data from MixRadio, comprising song downloads through Nokia cell phones in Great Britain from 2007 to 2014. We compared the ability of the standard susceptible–infectious–recovered (SIR) epidemic model and a phenomenological (spline) model to fit download time series of popular songs. We fitted these same models to simulated epidemic time series generated by the SIR model. Song downloads are captured better by the SIR model, to the same extent that actual SIR simulations are fitted better by the SIR model than by splines. This suggests that the social processes underlying song popularity are similar to those that drive infectious disease transmission. We draw conclusions about song popularity within specific genres based on estimated SIR parameters. In particular, we argue that faster spread of preferences for Electronica songs may reflect stronger connectivity of the ‘susceptible community’, compared with the larger and broader community that listens to more common genres.

## 1. Introduction

Music is ubiquitous in society; everyone listens to it and most people prefer certain styles [1]. This ubiquity results in an enormous variety of music and a huge number of songs for listeners to choose from. In spite of this abundance, a remarkably small number of popular songs are almost immediately recognizable to most people at a given time. How does a song become popular and how is it that certain songs become so much more popular than others? What are the underlying social mechanisms that drive these processes?

There are many similarities between the release of a new hit song and the outbreak of an infectious disease. When an infectious disease first enters a population, it is transmitted from person to person via social interactions. Prevalence eventually reaches a peak and then declines as the susceptible pool is exhausted and/or infectious individuals recover. After a new hit song is released, it also ‘spreads’ rapidly through a population, from person to person and through various media, eventually reaching some peak popularity and then diminishing in appeal [2]. At the end of a disease epidemic, a large proportion of the population will have been infected with the disease, whereas at the end of a hit song’s period of extreme popularity, a large proportion of the population will recognize that song.

Could the same social processes that facilitate spread of infectious disease in a population also drive song popularity? Popular songs are often described as ‘viral’ or ‘catchy’ as if they could ‘infect’ people; perhaps this description is more apt than has been previously recognized. In fact, the download time series for many popular songs that we examine in this study are similar in shape to time series for infectious diseases. This resemblance suggests that it is possible that there are social mechanisms underlying song popularity similar to the social mechanisms that drive the spread of an infectious disease, and has acted as our motivation to investigate standard epidemiological models as a tool to study song popularity.

Here, we consider how well a standard epidemiological model and a purely phenomenological spline can fit download time series for popular songs. For comparison, we also fit the same mechanistic and phenomenological models to stochastically simulated epidemic data. If song popularity is driven by a contagious process, then we would expect a mechanistic epidemic model to perform as well relative to a spline when the two models are applied to song download data as it does when they are applied to infectious disease data. If this is the case, then we can attach meaning to the epidemiological parameters estimated for popular songs based on disease transmission model fits and interpret these parameters to draw mechanistic conclusions about song popularity, which we cannot do with purely phenomenological models like splines.

We study data from a large and detailed database of song downloads from 2007 to 2014, a period when downloading (as opposed to streaming) was a primary method of music consumption (for further context on downloading versus streaming, see Aguiar [3]).

## 2. Background

### (a) Song popularity research

Song popularity has been the subject of much research. Some authors have sought to predict the peak and duration of a song’s popularity based solely on its previous popularity rankings [4,5]. Others have attempted to determine what musical features make a song popular [6–9]. While there may be specific musical characteristics that predict popularity, social processes also affect how a song gains popularity. Given that complex interactions undoubtedly exist between musical and social factors, disentangling the influence of each on a song’s popularity is a difficult task.

Previous research has found both support for [6,7] and evidence against [8,9] the idea that musical features can predict a song’s popularity. Nunes & Ordanini [7] used audio information to show that songs that were number 1 hits on the Billboard *Hot 100 Charts* in the past 55 years had distinctly different instrumentation than songs that never climbed above the 90th position on these charts. Dhanaraj & Logan [6] found that audio and lyric information about a song could each be used to generate better-than-random predictions about whether a song would be a hit. However, in their study of harmonic and timbral trends in the Billboard *Hot 100 Charts* over the past 50 years, Mauch *et al.* [9] found that the frequency of specific timbral characteristics cycled in the *Hot 100 Charts* as musical styles with different types of instrumentation came in and out of fashion. In addition, Pachet & Roy [8] failed to predict songs’ popularity based on audio characteristics, regardless of whether these characteristics were quantified from an audio signal or from human input.

Several studies [10–13] have found that information from social media sites, social music sites or peer-to-peer file-sharing networks can predict song popularity, which hints at the underlying social processes driving song popularity. Bischoff *et al.* [10] built a model that predicted song popularity based on various Last.fm tags relating to user listening habits and previous popularity of the artist in question; Koenigstein *et al.* [12] demonstrated that search queries from Gnutella could be used to predict a song’s peak position in the Billboard *Hot 100 Charts*. Schedl *et al.* [13] used Last.fm play-count data to predict popularity of artists in specific countries. They compared this method with predictions based on (i) user posts from Twitter, (ii) information from shared folders in Gnutella, and (iii) the number of pages returned by search engines that were related to an artist in a specific country. Kim *et al.* [11] also used Twitter posts to predict song popularity, finding that hashtags related to music listening behaviour of users could help forecast rankings of songs on Billboard charts. Zangerle *et al.* [14] extended this work to look at data over a longer time period. They found that although Twitter data alone were not sufficient to generate good song popularity predictions, using these data in multivariate predictive models significantly increased the models’ predictive ability.

Support for the idea that social interactions have a high impact on song popularity was presented in a study by Salganik *et al.* [15]. They played the same set of new music for several distinct groups of participants; song popularity was much less predictable, and between-song differences in popularity more extreme, when others’ opinions of songs were presented with the music.

Lastly, researchers have used neural imaging to examine the influence of a song’s overall popularity on adolescents’ rankings of that song [16]. The functional magnetic resonance imaging (fMRI) data collected in this study suggested that teenagers are more likely to change their evaluation of a song to more closely align with its overall popularity rating as a result of the anxiety created by a difference between their opinion and the opinion of others. Neural activity in specific regions of the brain while listening to songs significantly correlated with sales data for that song over the next 3 years, even though subjective ratings of the songs from participants did not [17].

### (b) Epidemiological modelling

**Figure 1.**
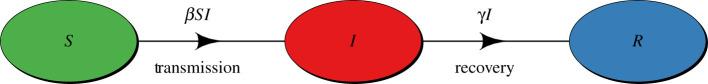
A flow chart representing how the susceptible–infectious–recovered (SIR) model tracks movement of individuals among the three disease state compartments. (Online version in colour.)

Infectious disease spread is commonly studied using a compartmental framework, in which individuals are classified according to disease state [18,19]. In typical situations where individuals acquire immunity after recovering, the simplest framework involves compartments containing ‘susceptible’, ‘infectious’ and ‘recovered’ individuals. This model is known as the SIR model (figure 1). The rate at which individuals move among the three compartments is given by a system of ordinary differential equations,

2.1a $$\begin{array}{rl} \frac{\text{d}S}{\text{d}t} & =-\beta SI, \end{array}$$

2.1b $$\begin{array}{rl} \frac{\text{d}I}{\text{d}t} & =\beta SI-\gamma I, \end{array}$$

2.1c $$\begin{array}{rl} \frac{\text{d}R}{\text{d}t} & =\gamma I, \end{array}$$

where β is the transmission rate and γ is the recovery rate. The SIR model also yields three slightly more intuitive parameters: the mean infectious period, 1/γ, the initial epidemic growth rate, r=β−γ, and the basic reproduction number, $R_{0}=\beta /\gamma$. $R_{0}$ is the expected number of individuals one infectious individual would infect in a wholly susceptible population. The expected final size Z of an epidemic can also be calculated based on $R_{0}$ [20,21] by solving for Z in the final size relation,

2.2 $$Z=1-{\text{e}}^{-R_{0}Z}.$$

Z is the expected proportion of the initially susceptible population that will have been infected over the entire course of the epidemic.

If the SIR model is interpreted in the context of popular songs, individuals are classified as being ‘susceptible to’, ‘infected with’ or ‘recovered from’ a song. The mean infectious period 1/γ measures the average time period for which an individual will continue to enjoy listening to a song, during which they may tell others about this song, thus ‘spreading’ it through the population. The basic reproduction number $R_{0}$ measures the average number of people in a wholly susceptible population who will be influenced to download a new song by one individual who is actively listening to and talking about this song. An individual has ‘recovered’ from a song when they are no longer actively listening to the song and spreading it to others.

When applied to infectious diseases, the SIR model can be used to draw a number of useful conclusions about an epidemic, such as how long an epidemic will last, what the final size of an epidemic will be and how quickly a disease will spread in a population. If it were successfully applied to song spread, these might be translated into conclusions about an epidemic of song downloads. For instance, it might be possible to estimate the duration of a song’s popularity, how many people in total will download it or how quickly it will become popular in a population.

This is not the first time epidemiological models have been applied to the dynamics of song popularity. A similar idea was employed by Tweedle & Smith? [22], who studied the effects of positive and negative media attention on ‘Bieber Fever’. However, while they were working with an epidemiological model, their study was entirely theoretical—they did not apply the ideas to any data. Rather than considering the general dynamics of song popularity, Tweedle & Smith? [22] focused on the excessive popularity of a particular artist within a specific demographic.

## 3. Description of the data

The database used for this study contains information on almost 1.4 billion individual song downloads. These data were obtained through a data-sharing agreement with MixRadio. Downloads occurred through Nokia cell phones in 33 countries over a 7 year period (2007–2014).^1^1In January 2015, the Nokia division responsible for online music became a separate entity under the name MixRadio; MixRadio ceased commercial operations in February 2016. Each data entry includes information about the download such as track title, artist name, artist genre classification and time of download. Various metadata about users are also housed in the database, including user ID, total number of downloads and user country. Because of its size and the nature of the data it contains, the database is an excellent tool for cultural and social investigations relating to music [23–26].

**Figure 2.**
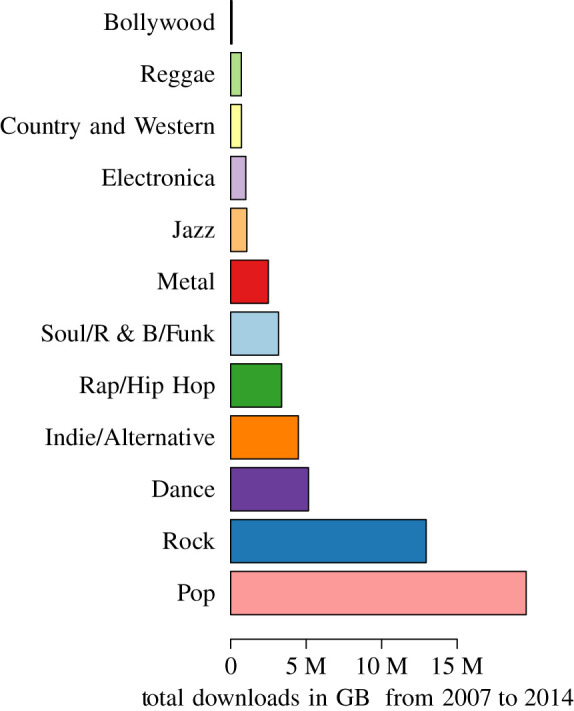
The proportion of total downloads by genre in Great Britain (GB) for the 12 genres that make up the list of the top 1000 most popular songs. The database contains information on 60 221 294 downloads by 552 784 users in GB. (Online version in colour.)

We focused our investigation on popular songs in Great Britain (GB), a country with an active downloading history. A list of the top 1000 songs ranked by number of downloads in GB was determined by considering downloads by all users in GB between 2007 and 2014 (figure 2). This selection provided a large sample of data (the database contains information on 60 221 294 downloads in GB by 552 784 users from 63 genres); focusing on a single country eliminated the issue of different countries adopting the MixRadio service at different times.

## 4. Methods

### (a) Top 1000 songs in Great Britain

#### (i) Data extraction and aggregation

The top 1000 songs in GB were defined as those with the most downloads in the database between 2007 and 2014. The database was queried using the open-source MySQL implementation of SQL [27]. The list of songs on which to test the SIR model was narrowed down by eliminating the 50 Christmas and holiday songs found in the top 1000 songs downloaded in GB. The time series for these songs display a pattern similar to seasonal epidemics, which the simple SIR model (equation (2.1)) cannot generate [18,19] (meaning that fitting the SIR model to these songs is not really a fair test). Minute-by-minute download counts for the remaining 950 songs were extracted, then aggregated at coarser time scales using the R statistical programming language [28]. The finest time scale used for most songs was daily since aggregating at time scales finer than this yielded noisy download time series and poor fits; however, some songs gained popularity so quickly that aggregating at the daily level obscured an initial increase in downloads. For these songs, the beginning of the time series was aggregated at a finer time scale than the rest to produce a time series that the SIR model could be fitted to; finer aggregation was conducted up to the point where the peak number of daily downloads occurred.

#### (ii) SIR fits

The SIR model was fitted to each resulting time series using the package fitsir in R; this package employs least-squares fitting to match solutions of the SIR model to a given time series—code available at https://github.com/bbolker/fitsir. To account for the possibility of multiple least-squares solutions, Latin hypercube sampling of the parameter space [29] was used to generate 100 possible SIR fits for each song and the best fit was then selected. The parameter space consisted of the transmission rate β, the recovery rate γ and the initial condition $\left(S_{0},I_{0},R_{0}\right)$, in which the initially recovered population is $R_{0}=0$, making the initially susceptible population $S_{0}$ and initially infectious population $I_{0}$ dependent on each other.

#### (iii) Spline fits

Cubic splines [30] were also fitted to each of the aggregated download time series in R. Three degrees of freedom (corresponding to one interior knot and two boundary knots) were used in all spline fits to match the three free parameters in our SIR model (the transmission rate β, the recovery rate γ and the initial condition $\left(S_{0},I_{0},R_{0}=0\right)$).

#### (iv) Goodness of fit

To compare the epidemiological model and the phenomenological model (cubic spline), we calculated the relative root mean squared error (RRMSE) to measure goodness of fit. This relative fit measure was calculated by finding the average relative distance between model trajectory and song download data point,

4.1 $$\sqrt{\text{mean}\left({\left(1-\frac{s}{c}\right)}^{2}\right)},$$

where c represents a download data point (i.e. the number of downloads that occurred at a particular point in time) and s represents a point on the SIR or spline model trajectory (i.e. the predicted number of downloads at the same time). A lower relative fit measure implies a better fit.

#### (v) Minimum $R_{0}$ and Z

A minimum possible basic reproduction number $R_{0}$ was also determined for songs released in GB. This was done using the final size formula (2.2) and the assumption that the final size Z for an epidemic of downloads for a particular song was the proportion of the initially susceptible population ($S_{0}$) that actually downloaded the song,

4.2 $$Z=\frac{\text{total downloads}}{S_{0}}.$$

The minimum possible final size Z for the country was calculated by taking the number of users in GB as the greatest possible susceptible population ($S_{0}$) and the smallest number of downloads for a song in the top 1000 most downloaded songs as the smallest possible number of ‘total downloads’. Since the final size formula (2.2) implies that Z strictly increases with $R_{0}$, it could then be used to determine the minimum possible $R_{0}$ (or $R_{\min}$) for GB, based on this minimum possible Z.

#### (vi) Criteria for satisfactory fits

At this point, the set of songs being considered was further restricted to those that yielded reasonable fits, meaning that any songs with an estimated $R_{0}$ less than $R_{\text{min}}$ were excluded from further analysis. In addition, visual inspection determined that songs for which the relative fit measure (see equation (4.1)) was greater than 11 gave a poor fit, so these songs were also excluded. Epidemiological parameter estimates were obtained based on the SIR fits for the remaining subset of songs and analysed.

#### (vii) Final size and initially susceptible population

A final size was determined for each individual song based on the estimated $R_{0}$ (using equation (2.2)). This final size Z is a proportion of the initially susceptible population $S_{0}$, i.e. the number of individuals initially susceptible to downloading a song. Equation (4.2) could therefore be used to calculate $S_{0}$ for each song based on knowledge of total downloads and estimated final size.

### (b) Simulated epidemic data

One thousand idealized infection curves were also generated using the Gillespie algorithm [31] to compute a stochastic solution for a given set of parameters. While it assumes that the intrinsic uncertainty comes entirely from an independent Poisson process (i.e. successive downloads are uncorrelated with each other—within the overall framework of the epidemic process, there is no additional heterogeneity such as ‘super-spreading’ events), this approach does generate reasonable levels of stochastic variation. The SIR model and cubic splines were fitted to each of the stochastic time series with the same methods used for song data. Goodness of fit was calculated using equation (4.1) as described above.

## 5. Results

### (a) SIR model fits and spline fits

**Figure 3.**
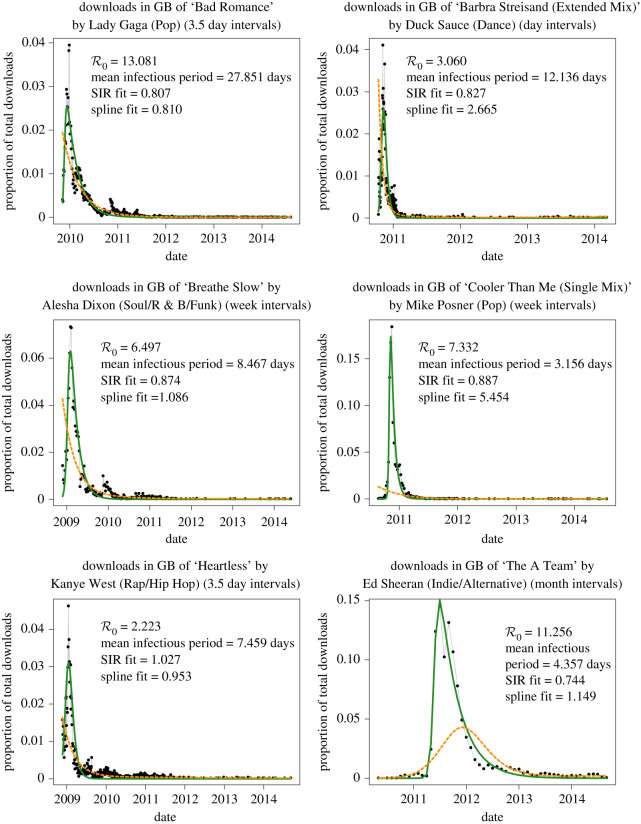
SIR and cubic spline fits for six of the songs considered in this study. Aggregated downloads are displayed as black dots connected by grey lines, with the time scale for aggregation printed in the title of each plot. The fitted epidemic curve is shown as a green solid line, and the fitted cubic spline is displayed as an orange dashed line. The relative fit measure (equation (4.1)) associated with the SIR model and the spline is printed in the top right of each plot, with lower numbers indicating a better fit. The basic reproduction number ($R_{0}$) and mean infectious period (1/γ) estimated based on the SIR fit are also displayed in the upper right of each plot. These are six songs for which the SIR model yielded a good fit. For songs like these, the SIR fitted curve generally follows the overall trajectory of the data more closely than the spline, even in many cases where the spline has a lower relative fit measure. (Online version in colour.)

**Figure 4.**
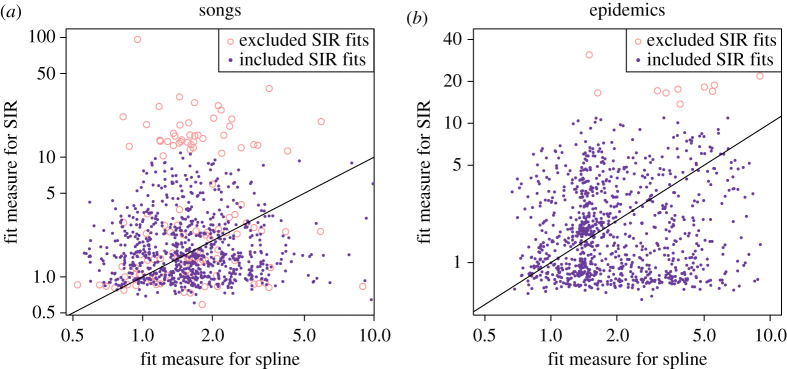
The relative fit measure (equation (4.1)) for the SIR model plotted against the relative fit measure for a cubic spline for all 950 songs in our sample set (*a*) and for all 978 simulated infection curves (*b*). Each point represents one song or simulated epidemic time series, with closed purple dots representing songs or epidemics that met our inclusion criteria and open pink circles representing songs or epidemics that did not. Lower fit measures represent better fits; thus points that fall below the plotted y=x line represent cases where the SIR model yielded a better fit than a cubic spline. (Online version in colour.)

**Figure 5.**
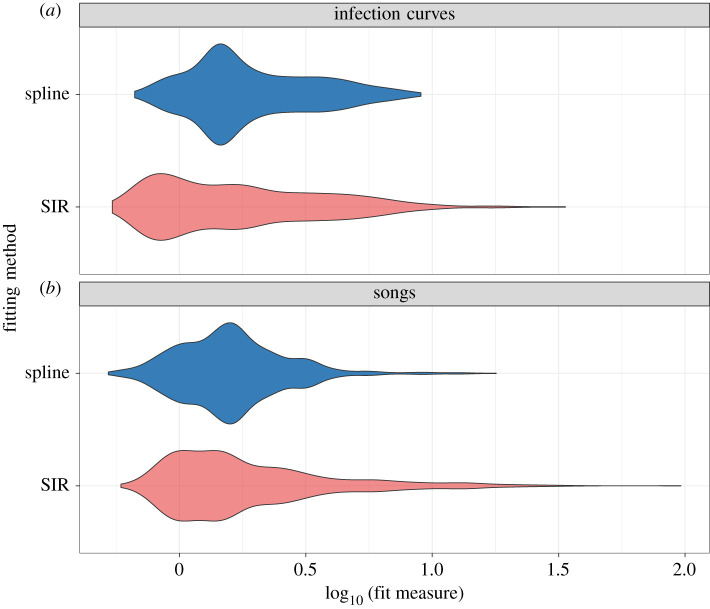
Distribution of the relative fit measure (equation (4.1)) for the SIR model and a cubic spline applied to the 950 songs in our sample set (*b*) and applied to the 978 simulated infection curves (*a*). (Online version in colour.)

Of the 950 songs in our sample set, 828 (87.2%) met our fitting criteria, i.e. they were well captured by the basic SIR model. Figure 3 compares the SIR model fit with the cubic spline fit for six of the songs that were well fitted by the SIR model. A cubic spline yielded a better fit for 484 of the 950 songs in our sample (50.9%); of the 828 songs that were well captured by the SIR model, 422 were better fitted by the SIR model than by the spline (51%; see figure 4). For the song download curves, the median relative fit measure for the SIR model was 1.50. This number was slightly lower (i.e. better) than the median relative fit measure for cubic splines, which was 1.85; however, for SIR fits, the distribution of the relative fit measure had a long tail (figure 5). The SIR model often yielded a visually better fit for songs (i.e. a fit that better followed the main rise and fall of the download trajectory) even in cases where the spline yielded a better relative fit measure; this was particularly true for songs that had a very good SIR fit.

Similar trends were seen in the performance of the SIR model against cubic splines when applied to simulated epidemic data, Of the 978 epidemic curves in our sample, 967 (98.9%) were well captured by the SIR model, based on our fitting criteria (they had a relative fit measure of less than 11). Of the entire set of 978 simulations, a cubic spline yielded a better fit for 462 of them (47.2%); of the 967 well captured curves, the SIR model yielded a better fit than a spline for 516 of them (53.4%; see figure 4). As with song download curves, the median relative fit measure for the SIR model applied to epidemic curves (1.53) was slightly lower than the median relative fit measure for cubic splines (1.62), with the SIR model fits again displaying a long tail in their relative fit measure distribution (figure 5).

These results show that the SIR model performed very similarly against cubic splines when fitted to songs as it did when fitted to epidemic curves (figure 5). The SIR model is a good representation of the mechanisms that drive infectious disease spread; since it appears to fit song download data as well as it fits simulated SIR epidemic data, it is reasonable to propose that it captures some underlying social drivers of song popularity.

### (b) Estimated and derived parameters

For the song download time series, the distributions of all estimated (i.e. fitted) or derived (calculated from the estimated parameters) parameters were explored. Once we concluded that the SIR model was a reasonable approximation of the scenario being studied, we could draw conclusions based on the interpretation of these parameters in the context of song popularity. Interpretable parameters are one major advantage of a mechanistic model over a phenomenological model such as a spline. Table 1 gives the median estimated values for some key epidemiological parameters across the genres in our sample.

**Table 1.** Median epidemiological parameter values, by genre, for the 828 songs in our dataset that met our good fit criteria. Basic reproduction number $R_{0}$, mean infectious period 1/γ (in days), transmission rate β (per day), initial epidemic growth rate r=β−γ (per day) and doubling time (days).

genre	no. songs	$R_{0}$	1/γ	β	r	$\frac{\ln2}{r}$
Bollywood	1	25.01	45.25	0.55	0.53	1.30
Country and Western	5	4.77	33.60	0.30	0.24	2.95
Dance	106	2.84	7.49	0.37	0.23	3.04
Electronica	11	3430.01	199.10	19.50	19.49	0.04
Indie/Alternative	68	25.20	39.75	6.89	3.06	0.23
Metal	10	3.65	9.08	0.37	0.25	2.78
Pop	336	35.03	20.62	4.48	2.33	0.30
Rap/Hip Hop	104	310.94	93.25	6.99	3.40	0.20
Reggae	4	5.64	10.76	0.53	0.43	1.61
Rock	109	129.33	16.04	11.91	11.80	0.06
Soul/R&B/Funk	74	31.27	42.10	1.41	0.99	0.70

**Figure 6.**
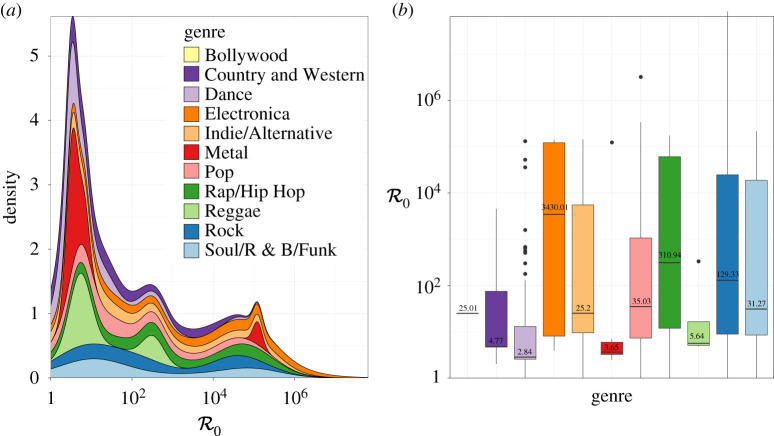
Distribution across genre of the basic reproduction number $R_{0}$ estimated from the SIR model fitted to the 828 songs in our sample set. Genres are colour-coded as in figure 2. There are 222 songs with $R_{0}>1000$ and seven songs with $R_{0}>10^{6}$. In the stacked density plot in (*a*), there are three modes at $R_{0}=31$, 595 and 40 955. The sampled number of songs varies greatly among the different genres, from 1 to 336 (table 1). (Online version in colour.)

The basic reproduction number $R_{0}$ has a natural interpretation in the context of songs; it tells us—in a fully ‘susceptible’ population—how many people would be influenced to download a song by an ‘infectious’ individual actively spreading that song (e.g. by talking about the song, playing it, sharing it on social media or requesting it on the radio). Figure 6 shows the distribution of $R_{0}$ for the well-fitted songs from each genre in our sample set. The median $R_{0}$ varies substantially among genres, with Dance having the lowest median $R_{0}$ of 2.84 and Electronica having the highest median $R_{0}$ of 3430. One might expect Pop to have the highest $R_{0}$, since this genre of music is the most widely played on the radio, at public events, on TV and in movies; however, its median $R_{0}$ of 35 is far outstripped by genres like Electronica, Rock (129) and Rap/Hip Hop (311).

**Table 2.** Median values by genre of calculated initially susceptible population $S_{0}$, downloads and final size Z for the 828 songs in our dataset.

genre	no. songs	calculated $S_{0}$	downloads	Z
Bollywood	1	4237	4237	1.000
Country and Western	5	4048	4048	0.991
Dance	106	6363	5759	0.929
Electronica	11	4737	4710	1.000
Indie/Alternative	68	6706	6326	1.000
Metal	10	5161	4922	0.971
Pop	336	5592	5317	1.000
Rap/Hip Hop	104	5268	5248	1.000
Reggae	4	4147	4130	0.996
Rock	109	5182	4979	1.000
Soul/R&B/Funk	74	5946	5752	1.000

**Figure 7.**
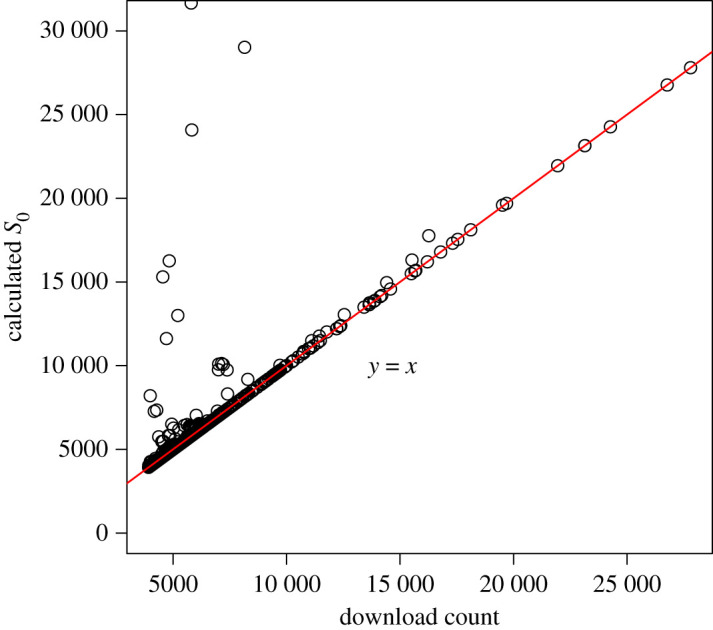
Estimated initial susceptible population $S_{0}$ versus total number of downloads for each of the 828 songs in the database that met our inclusion criteria. The red line (y=x) runs through the set of points for which final size Z is very close to 1. Sixty-two songs lie outside the bounds of this plot because their calculated $S_{0}$ value is too great. (Online version in colour.)

When looking at individual songs, the estimated initially susceptible population $S_{0}$ for a song (based on the song’s fitted epidemic curve) was almost always less than the total download count for that song. Since the total download count should not exceed the initially susceptible population, we instead examined the $S_{0}$ values calculated using equation (4.2) (based on applying the final size relation equation (2.2) to obtain Z from the estimated $R_{0}$ and using the observed download count, as described in §4). Table 2 gives the median values by genre for download count, estimated final size and calculated initially susceptible population. The final size Z was usually close to 1, with 727/828 (87.8%) songs having Z>0.9 and 640/828 (77.3%) having Z>0.99, meaning that the calculated $S_{0}$ was generally very close to the download count. Since $S_{0}=1/Z\times \text{(total downloads) }$ (equation (4.2)), the songs for which Z is close to 1 form a straight line with slope slightly greater than 1 on the graph of calculated $S_{0}$ versus download count (figure 7). However, Z being nearly constant with values close to 1 for most of the songs in our sample set does not mean that $R_{0}$ was nearly constant (figure 6 shows the wide distribution of estimated $R_{0}$ values). This is because, according to equation (2.2), Z→1 quite quickly as $R_{0}$ increases (Z=0.98 already for $R_{0}=4$). The high estimates of $R_{0}$ for most songs (655/828 songs had an estimated $R_{0}>4$) explain why most estimates of final size Z were quite close to 1.

## 6. Discussion

The SIR model describes song download trends for popular songs well; it may be a good representation of the processes driving song popularity. Our results support the idea that the model could capture an underlying ‘song transmission’ mechanism or a contagious process that drives song popularity. Since the SIR model is mechanistic, the parameters that were estimated from SIR fits to song download data (table 1) and derived from those estimates (table 2) can provide insight into song popularity based on the mechanistic interpretation of these parameters. In particular, we can make inferences about the distinguishing characteristics of fans (susceptible populations) of different genres.

Although the median values for many of the estimated parameters differ significantly between genres (tables 1 and 2), median final size Z is quite consistent. Most songs infect their entire susceptible population, meaning that Z (and therefore median Z) is almost always nearly 1.

The median $R_{0}$, however, varies substantially between genres. For Pop and Electronica, this difference between median $R_{0}$ tells an interesting story. Although we might expect Pop to have the highest median $R_{0}$ of all genres because of its name, it is in fact Electronica, a more niche genre, that holds this record by two orders of magnitude (table 1). However, this high median $R_{0}$ does not seem to correspond to many more downloads of Electronica songs than Pop songs; in fact, the median download count is relatively consistent across genres—a result we might expect so long as the songs for each genre were evenly distributed through the list of top 1000 most popular songs based on download count. Instead, we see Electronica’s high $R_{0}$ values manifest in shorter, faster epidemics; the download time series for Electronica songs show the majority of downloads happening in a shorter time period than Pop songs, meaning that these songs appear to gain popularity faster than those in other genres, and to burn through their susceptible populations more quickly. Indeed, the median initial growth rate r is substantially higher for Electronica songs than for Pop songs, and the median doubling time is substantially shorter (table 1). These observations support previous work which showed that the pattern of downloads differs for songs depending on their genre [24,25].

What does this pattern tell us about fans of the different genres and how songs from each genre are transmitted? Perhaps fans of Electronica transmit these songs more actively or more effectively. The social network of Electronica fans might be more strongly connected than fan communities of other music genres such as Pop. Electronica fans may be more passionate about their favourite songs and bands than Pop fans, and therefore talk about and promote their favourite songs more. Perhaps Pop, being a more mainstream genre, is spread chiefly through more passive means like the radio. Such mechanistic hypotheses could not be derived from the analysis of a phenomenological model like our cubic spline fits.

This comparison between Pop and Electronica can be taken further if we consider susceptible populations. In our context, the susceptible population is defined as the group of individuals who may download a song if exposed to it. Since most individual songs had a final size Z very close to 1, their download count tended to be very close to their calculated susceptible population. Interestingly, the median download count and median susceptible population do not vary much between genres (table 2). This result again defies expectations about the Pop genre, namely that Pop songs would have the largest susceptible populations since it is literally ‘popular music’. It is more likely instead that more people are exposed to Pop (and possibly that a higher number of people will tolerate listening to Pop), whereas only the susceptible population is exposed to a more niche genre like Electronica.

### (a) Future research

Although many people now consume music via streaming services, this study uses data from a large and detailed database of song *downloads*. Due to the nature of the data available, we have focused our analysis on song download behaviour. With a different dataset, the same type of analysis could be applied to streaming data, which might offer different information.

The SIR model is one of the most basic epidemiological models; as such, it neglects many aspects of disease transmission. Although our results show that the model describes song download dynamics well, it has too little structure to represent all the nuances of a song spreading through a population. Fitting other disease models, with more biological structure, to song download data might illuminate the most important processes driving ‘song transmission’. Details worth considering include vital dynamics (‘births’ and ‘deaths’, i.e. users who join or leave a downloading service like MixRadio), the role that the conditions under which a song is released play in its success or failure (potentially analogous to heterogeneity in transmission, which has been included in simple disease models by modifying the transmission term to be nonlinear in S and/or I [32,33]), or the effect that social structure and human behaviour have on disease dynamics [34]. A reservoir model, such as those used to model spread of waterborne disease, might capture the influence of the radio and streaming services on the spread of a song [35,36]. A disease model that incorporated an initial pulse of infection might better represent the effect of mass media and marketing promotion that some songs receive. Imperfect vaccination models could be used to model the changing musical preferences of individuals within a population [37]. With access to live stream data, rather than just download data, one could apply a model that accounts for decay in immunity to the dataset, such as the SIS (susceptible–infectious–susceptible) or SIRS (susceptible–infectious–recovered–susceptible) model [38], or models with both decay of immunity and nonlinear incidence [32,33].

It might also be fruitful to pursue the concept of super-spreading in the context of song download epidemics, i.e. the idea that the basic reproduction number $R_{0}$ can vary substantially within a population when certain individuals have a higher degree of infectiousness than others [39]. In the context of songs, super-spreaders might be people who express their opinions of a song much more often and readily, more strongly/passionately and/or through a widely accessed social media platform. A potential avenue of future research would be to identify characteristics of song super-spreaders that would be detectable in the data.

The usual implementations of cubic splines result in smooth curves that maximize some measure of fit to the data, but they are not constrained to produce only unimodal curves (i.e. curves with an intermediate maximum, the typical shape of an epidemic curve). In our study, this means that a cubic spline that yielded a very good fit (according to our criterion equation (4.1)) might not follow the shape of the data it was fitting (e.g. ‘Bad Romance’, ‘Breathe Slow’ and ‘Heartless’ in figure 3). It would be interesting to investigate the performance of phenomenological models that have a built-in assumption of unimodality (such as those in [40,41]).

## 7. Conclusion

This study has explored the utility of a mechanistic epidemic model (the SIR model) for describing song popularity by comparing its ability to capture song download patterns against that of a phenomenological model (cubic spline), and comparing this with each model’s ability to capture infectious disease spread patterns. The SIR model performed similarly to the cubic spline both when fitted to song download data and when fitted to simulated epidemic curves, which is what we would expect if popular songs are indeed ‘infectious’. Thus, our results indicate that song popularity may be driven by an underlying contagious process. Since the SIR model is mechanistic, we were able to make mechanistic inferences about song popularity based on parameters estimated from fits. Specifically, we drew conclusions about how the downloading and music-sharing behaviours of music fans may differ by genre.

To our knowledge, our analysis is the first comparison of the ability of splines with mechanistic epidemiological models to fit epidemic curves. When presented with infectious disease data, it is natural to consider mechanistic transmission models rather than phenomenological models. By contrast, if one is merely considering the possibility that some observed data were generated by a contagious process, then it is natural to consider a variety of models. Although the focus of this study is on song popularity, this work has an important methodological theme that has broader significance: to infer that ‘mechanism X’ was likely involved in generating a given dataset, the key question to ask is not ‘Does model X do better than model Y?’ but ‘Does model X perform better than model Y to the same extent that it does for data known to have been generated by model X?’

We have shown that epidemic models offer a powerful tool for analysing music downloading trends and studying the mechanisms that drive song popularity. Applying some of the many possible extensions to the simple SIR model could help us to learn more about how songs become popular and how the mechanisms that drive song popularity relate to those that drive disease epidemics.

## Data accessibility

The original database containing individual downloads from individual users is proprietary. The aggregated data that were used for analysis and to make the figures in this paper (including fit measures and estimated/derived parameters) have been provided, along with a sample of song download proportion data. The data and scripts used for analysis are hosted in the github repository https://github.com/drosati/SongDownloadEpidemics [42].

## References

[1] Brown D. 1991 Human universals. New York, NY: McGraw-Hill.

[2] Dewan S, Ramaprasad J. 2014 Social media, traditional media, and music sales. MIS Q. **38**, 101-122.

[3] Aguiar L. 2017 Let the music play? Free streaming and its effects on digital music consumption. Inf. Econ. Policy **41**, 1-14. (10.1016/j.infoecopol.2017.06.002)

[4] Bradlow ET, Fader PS. 2001 A Bayesian lifetime model for the ‘Hot 100’ Billboard songs. J. Am. Stat. Assoc. **96**, 368-381. (10.1198/016214501753168091)

[5] Chon SH, Slaney M, Berger J. 2006 Predicting success from music sales data: a statistical and adaptive approach. In *Proc. 1st ACM Workshop on Audio and Music Computing Multimedia, Santa Barbara, CA, 27 October 2006*, pp. 83–88. New York, NY: ACM.

[6] Dhanaraj R, Logan B. 2005 Automatic prediction of hit songs. In *Proc. Int. Symp. on Music Information Retrieval, London, UK, 11–15 September 2005*, pp. 488–491. London, UK: Queen Mary University of London.

[7] Nunes JC, Ordanini A. 2014 I like the way it sounds: the influence of instrumentation on a pop song’s place in the charts. Music. Sci. **18**, 392-409. (10.1177/1029864914548528)

[8] Pachet F, Roy P. 2008 Hit song science is not yet a science. In *Proc. Int. Symp. on Music Information Retrieval, Philadelphia, PA, 14–18 September 2008*, pp. 355–360.

[9] Mauch M, MacCallum RM, Levy M, Leroi AM. 2015 The evolution of popular music: USA 1960–2010. R. Soc. Open Sci. **2**, 150081. (10.1098/rsos.150081) 26064663 PMC4453253

[10] Bischoff K, Firan CS, Georgescu M, Nejdl W, Paiu R. 2009 Social knowledge-driven music hit prediction. In *Advanced data mining and applications* (eds R Huang, Q Yang, J Pei, J Gama, X Meng, X Li), pp. 43–54. Berlin, Germany: Springer.

[11] Kim Y, Suh B, Lee K. 2014 Nowplaying the future billboard: mining music listening behaviors of Twitter users for hit song prediction. In *SIGIR '14: The 37th International ACM SIGIR Conference on Research and Development in Information Retrieval, Gold Coast, Queensland, Australia, 11 July 2014*, pp. 51–56. New York, NY: Association for Computing Machinery.

[12] Koenigstein N, Shavitt Y, Zilberman N. 2009 Predicting billboard success using data-mining in P2P networks. In *ISM’09, 11th IEEE Int. Symp. on Multimedia*, pp. 465–470. New York, NY: IEEE.

[13] Schedl M, Pohle T, Koenigstein N, Knees P. 2010 What’s hot? Examining country-specific artist popularity. In *Proc. of the 11th Society for Music Information Retrieval Conference (ISMIR 2010), Utrecht, The Netherlands, 9–13 August 2010*, pp. 117–122.

[14] Zangerle E, Pichl M, Hupfauf B, Specht G. 2016 Can microblogs predict music charts? An analysis of the relationship between #nowplaying tweets and music charts. In *Proc. 17th Int. Soc. for Music Information Retrieval Conf., ISMIR 2016, New York, NY, 7–11 August 2016*, pp. 365–371.

[15] Salganik MJ, Dodds PS, Watts DJ. 2006 Experimental study of inequality and unpredictability in an artificial cultural market. Science **311**, 854-856. (10.1126/science.1121066) 16469928

[16] Berns GS, Capra CM, Moore S, Noussair C. 2010 Neural mechanisms of the influence of popularity on adolescent ratings of music. Neuroimage **49**, 2687-2696. (10.1016/j.neuroimage.2009.10.070) 19879365 PMC2818406

[17] Berns GS, Moore SE. 2011 A neural predictor of cultural popularity. J. Consum. Psychol. **22**, 154-160. (10.1016/j.jcps.2011.05.001)

[18] Anderson RM, May RM. 1991 Infectious diseases of humans: dynamics and control. Oxford, UK: Oxford University Press.

[19] Hethcote HW. 2000 The mathematics of infectious diseases. SIAM Rev. **42**, 599-653. (10.1137/S0036144500371907)

[20] Kermack WO, McKendrick AG. 1927 A contribution to the mathematical theory of epidemics. Proc. R. Soc. Lond. A **115**, 700-721. (10.1098/rspa.1927.0118)

[21] Ma J, Earn DJD. 2006 Generality of the final size formula for an epidemic of a newly invading infectious disease. Bull. Math. Biol. **68**, 679-702. (10.1007/s11538-005-9047-7) 16794950 PMC7088645

[22] Tweedle V, Smith R. 2012 A mathematical model of Bieber fever: the most infectious disease of our time? In *Understanding the dynamics of emerging and re-emerging infectious diseases using mathematical models* (eds S Mushayabasa, CB Bhunu), pp. 157–177. Kerala, India: Transworld Research Network.

[23] Bansal J, Woolhouse M. 2015 Predictive power of personality on music-genre exclusivity. In *Proc. 16th Int. Soc. for Music Information Retrieval Conf., Malaga, Spain, 26–30 October 2015*, pp. 652–658.

[24] Woolhouse MH, Renwick J, Tidhar D. 2014 Every track you take: analysing the dynamics of song and genre reception through music downloading. Digit. Stud./Le Champ Numer. **5**. (10.16995/dscn.51)

[25] Woolhouse MH, Renwick J. 2016 Generalizing case-based analyses in the study of global music consumption. Digit. Stud./Le Champ Numer. **5**. (10.16995/dscn.25)

[26] Barone MD, Bansal J, Woolhouse MH. 2017 Acoustic features influence musical choices across multiple genres. Front. Psychol. **8**, 931. (10.3389/fpsyg.2017.00931) 28725200 PMC5495864

[27] Weinberg P, Groff J, Oppel A, Davenport A. 2010 SQL, the complete reference. New York, NY: McGraw-Hill.

[28] R Core Team. 2016 *R: a language and environment for statistical computing*. Vienna, Austria. See www.R-project.org.

[29] Raue A *et al.* 2013 Lessons learned from quantitative dynamical modeling in systems biology. PLoS ONE **8**, e74335. (10.1371/journal.pone.0074335) 24098642 PMC3787051

[30] Venables W, Ripley BD. 2002 Modern applied statistics with S, 4th edn. New York, NY: Springer.

[31] Gillespie DT. 1976 A general method for numerically simulating the stochastic time evolution of coupled chemical reactions. J. Comput. Phys. **22**, 403-434. (10.1016/0021-9991(76)90041-3)

[32] Liu WM, Levin SA, Iwasa Y. 1986 Influence of nonlinear incidence rates upon the behavior of SIRS epidemiological models. J. Math. Biol. **23**, 187-204. (10.1007/BF00276956) 3958634

[33] Liu WM, Hethcote HW, Levin SA. 1987 Dynamical behavior of epidemiological models with nonlinear incidence rates. J. Math. Biol. **25**, 359-380. (10.1007/BF00277162) 3668394

[34] Funk S, Salathe M, Jansen VAA. 2010 Modelling the influence of human behaviour on the spread of infectious diseases: a review. J. R. Soc. Interface **7**, 1247-1256. (10.1098/rsif.2010.0142) 20504800 PMC2894894

[35] Tien JH, Earn DJD. 2010 Multiple transmission pathways and disease dynamics in a waterborne pathogen model. Bull. Math. Biol. **72**, 1506-1533. (10.1007/s11538-010-9507-6) 20143271

[36] Tien JH, Poinar HN, Fisman DN, Earn DJD. 2011 Herald waves of cholera in nineteenth century London. J. R. Soc. Interface **8**, 756-760. (10.1098/rsif.2010.0494) 21123253 PMC3061096

[37] Gumel AB, McCluskey CC, van den Driessche P. 2006 Mathematical study of a staged-progression HIV model with imperfect vaccine. Bull. Math. Biol. **68**, 2105-2128. (10.1007/s11538-006-9095-7) 16868850

[38] Dushoff J, Plotkin JB, Levin SA, Earn DJD. 2004 Dynamical resonance can account for seasonality of influenza epidemics. Proc. Natl Acad. Sci. USA **101**, 16 915-16 916. (10.1073/pnas.0407293101) 15557003 PMC534740

[39] Lloyd-Smith JO, Schreiber SJ, Kopp PE, Getz WM. 2005 Superspreading and the effect of individual variation on disease emergence. Nature **438**, 355-359. (10.1038/nature04153) 16292310 PMC7094981

[40] Koellman C, Bornkamp B, Ickstadt K. 2014 Unimodal regression using Bernstein-Schoenberg splines and penalties. Biometrics **70**, 783-793. (10.1111/biom.12193) 24975523

[41] Koellmann C. 2016 uniReg: unimodal penalized spline regression using B-splines; R package version 1.1. See https://CRAN.R-project.org/package=uniReg.

[42] Rosati D, Woolhouse M, Bolker B, Earn D. 2021 Data from: drosati/SongDownloadEpidemics: Song Download Epidemics (SongDownloadEpidemics). Zenodo. (10.5281/zenodo.5496169) PMC8455174 35153583

